# Changes in Physical Activity, Heart Rate, and Sleep Measured by Activity Trackers During the COVID-19 Pandemic Across 34 Countries: Retrospective Analysis

**DOI:** 10.2196/68199

**Published:** 2025-04-04

**Authors:** Bastien Wyatt, Nicolas Forstmann, Nolwenn Badier, Anne-Sophie Hamy, Quentin De Larochelambert, Juliana Antero, Arthur Danino, Vincent Vercamer, Paul De Villele, Benjamin Vittrant, Thomas Lanz, Fabien Reyal, Jean-François Toussaint, Lidia Delrieu

**Affiliations:** 1 Institute for Research in bioMedicine and Epidemiology of Sport Université Paris Cité Paris France; 2 INSEP (Institut National du Sport, de l'Expertise et de la Performance) Paris France; 3 Residual Tumor and Response to Treatment Laboratory, Translational Research Department INSERM, U932 Immunity and Cancer, Institut Curie Université Paris Cité Paris France; 4 Withings Issy-les-Moulineaux France; 5 Department of Anesthesiology Clinique de la Sauvegarde Lyon France; 6 Department of Surgical Oncology Institut Curie Université Paris Cité Paris France; 7 CIMS (Center for Investigations in Medicine and Sports) Hôtel-Dieu, Assistance Publique – Hôpitaux de Paris Paris France

**Keywords:** Covid-19, pandemic, physical activity, step, activity tracker, public health, Withings, heart rate, wearable sensors, sleep duration, sleep quality, pre-pandemic, public health, sedentary behavior

## Abstract

**Background:**

The COVID-19 pandemic disrupted behavior within populations, affecting physical activity (PA), heart rate (HR), and sleep characteristics in particular. Activity trackers provide unique insights into these changes, enabling large-scale, real-time monitoring.

**Objective:**

This study aims to analyze the associations between the features of the COVID-19 pandemic worldwide and PA, HR, and sleep parameters, using data collected from activity trackers over a 3-year period.

**Methods:**

We performed a retrospective analysis using anonymized data collected from the 208,818 users of Withings Steel HR activity trackers, spanning 34 countries, over a 3-year period from January 2019 to March 2022. Key metrics analyzed included daily step counts, average heart rate, and sleep duration. The statistical methods used included descriptive analyses, time-trend analysis, and mixed models to evaluate the impact of restriction measures, controlling for potential confounders such as sex, age, and seasonal variations.

**Results:**

We detected a significant decrease in PA, with a 12.3% reduction in daily step count (from 5802 to 5082 steps/d) over the 3 years. The proportion of sedentary individuals increased from 38% (n=14,177) in 2019 to 52% (n=19,510) in 2020 and remained elevated at 51% (n=18,972) in 2022, while the proportion of active individuals dropped from 8% (n=2857) to 6% (n=2352) in 2020 before returning to 8% (n=2877) in 2022. In 2022, the global population had not returned to prepandemic PA levels, with a noticeable persistence of inactivity. During lockdowns, HR decreased by 1.5%, which was associated with lower activity levels. Sleep duration increased during restrictions, particularly in the countries with the most severe lockdowns (eg, an increase of 15 min in countries with stringent measures compared to 5 min in less restricted regions).

**Conclusions:**

The sustained decrease in PA and its physiological consequences highlight the need for public health strategies to mitigate the long-term effects of the measures taken during the pandemic. Despite the gradual lifting of restrictions, PA levels have not fully recovered, with lasting implications for global health. If similar circumstances arise in the future, priority should be given to measures for effectively increasing PA to counter the increase in sedentary behavior, mitigate health risks, and prevent the rise of chronic diseases.

## Introduction

On March 11, 2020, the Director-General of the World Health Organization (WHO) declared a global pandemic of COVID-19. By April 2023, more than 764 million cases had been confirmed and 6.9 million deaths had been reported worldwide [1]. Governments in countries such as France, Italy, Spain, the United Kingdom, and the United States implemented various nonpharmaceutical interventions, including social distancing, quarantines, lockdowns, and the closure of offices and schools. These measures significantly disrupted daily routines, leading to an approximate 28% decrease in physical activity (PA) levels globally, an increase in sedentary behaviors, and substantial challenges to mental health [2-8].

A systematic review highlighted the beneficial effects of PA on physical and mental health during the first year of the COVID-19 pandemic [9]. The review found that engaging in regular PA helped to mitigate the negative impact of pandemic-induced stress [9]. In addition, inactive individuals were reported to have lower well-being scores and higher levels of depression and anxiety than moderately active and active individuals. A large-scale meta-analysis of data for 1,853,610 adults revealed that the rates of severe COVID-19 were 34% lower, the risk of hospitalization was 36% lower, and COVID-related mortality was 43% lower in participants regularly engaging in PA than in their inactive peers [10].

Even before this pandemic, the WHO and other health bodies had issued warnings about the global decline in PA, particularly in high-income countries, and strong gender, territorial, and financial inequalities [11,12]. Insufficient activity increased by 5% (from 31.6% to 36.8%) in high-income countries between 2001 and 2016, indicating a preexisting downward trend [13]. This decline has been linked to increases in the risks of chronic diseases, such as cancers, cardiovascular diseases, and diabetes, by approximately 20%-30% [14-16]. Conversely, one study showed that increasing the amount of moderate-intensity PA by 10, 20, or 30 minutes per day is associated with decreases in annual mortality of 6.9%, 13%, and 16.9%, respectively [17]. The pandemic has drawn further attention to this public health issue, as PA levels plummeted [18] and sports participation dropped by 20% in France alone during the first lockdown of 2020 relative to the corresponding period in 2019 [19]. In addition to health consequences, a systematic review has shown that PA is associated with higher health care costs, further underscoring the economic burden of insufficient PA [20,21]. In response, the WHO has established a global action plan aiming to decrease rates of physical inactivity by 15% by 2030 through strategic objectives and specific actions [22].

Advanced digital health technologies, including activity trackers, have emerged as possible tools for monitoring and potentially enhancing PA and health metrics, including step counts, heart rate (HR), and sleep patterns [23]. Such devices provide regular feedback, which may be instrumental in improving PA levels and overall health [24-29].

In this study, we investigated the changes in PA, sleep duration, and heart rate among users of wearable Withings Steel HR activity trackers from 34 countries during successive periods of the pandemic in which major public health policies were implemented. By measuring the variations of risk factors and making use of extensive user-generated data, in a real-life context, this study addressed the broader implications of the pandemic for lifestyle changes and health.

## Methods

### Study Design

We performed a retrospective analysis of 3 datasets (Step, HR, and Sleep datasets) collected from Withings Steel HR activity trackers. We included data from individuals with at least 1000 measures of the studied variables over the 3-year period, and compared countries with at least 100 individuals. This study adhered to the STROBE (Strengthening the Reporting of Observational Studies in Epidemiology) guidelines (STROBE checklist provided in Multimedia Appendix 1).

### Reported Outcomes for the Study Population

#### Overview

All self-reported data were provided at the time of registration to use the application. We collected available data from January 2019 onwards.

#### Sociodemographic and Anthropometric Data

The sociodemographic data studied included sex, age, and country of residence. The anthropometric data studied included height (cm), weight (kg), and BMI (weight divided by height squared). Participants were classified on the basis of BMI as being underweight (<18.5 kg/m^2^), normal weight (18.5-24.9), overweight (25-29.9), or obese (≥30), in accordance with WHO guidelines [30].

### Wearable Data Collection

#### Number of Steps and Minutes of Activity

The activity tracker collects step data around the clock over 24-hour periods, with a reset occurring at midnight. The number of steps is aggregated for the preceding 24-hour period during the daily reset, and the median number of daily steps is then calculated for each individual. Based on a compendium, the tracker’s algorithm detects activity throughout the day and categorizes it into durations of low-, moderate-, and high-intensity activities [31]. Workouts may be self-reported by users.

#### HR Measurement

HR is measured in beats per minute, with one measurement every 10 minutes. The dataset was composed of aggregated data, with mean values calculated separately for each day and night. We considered the HR averaged during sleep as the resting HR.

#### Sleep

The sleep data recorded include bedtime and wake-up time, time taken to fall asleep, total sleep duration, duration of deep, and light sleep, and the number of sleep interruptions.

### Wearable Data Processing and Missing Values

We analyzed data for individuals in 5 age classes (18-24, 25-39, 40-54, 55-64, and ≥65 y) and four BMI categories: underweight, normal, overweight, and obese [30]. Outliers were removed using the Z-scores at the intraindividual level. A threshold of 4 was chosen, as values beyond this range are typically considered extreme in statistical analysis, reducing the influence of outliers while retaining most of the data. This choice was particularly appropriate given that step count data often do not follow a normal distribution, necessitating a more flexible approach to outlier detection [32].

The number of steps ranged from 0 to 60,000 steps. A number of steps equal to 0 indicates that the user connected their activity tracker to the application but did not wear the activity tracker. Each line for which 0 steps were recorded was excluded from further analysis. Using the median number of steps in 2019 (reference year), we classified individuals according to the Tudor-Locke categories: sedentary (<5000 steps), low active (5000-7500 steps), somewhat active (7500-10,000 steps), and active (>10,000 steps) [33].

### Statistical Analysis

#### PA Levels (Step Dataset)

We first calculated the median daily step count for each individual across the entire follow-up period. The median values were chosen to minimize the influence of extreme values. We then computed the mean of these individual median step counts for each country and each year to assess overall trends.

Longitudinal changes in PA were analyzed using mixed-effects models, where the median daily step count was the dependent variable. The model included fixed effects (β), which represented factors influencing changes in step count, such as year, season, weekday, and biological gender. Random effects (u) accounted for individual variability and temporal factors, capturing within-subject correlations. The model equation was as follows:

where *Y_i_* represents the median number of steps for individual *i*, *α* is the coefficient associated with the *X* variable, *β* represents the intercept, *β_i_* represents the random effect of the intercept of individual *i*, and ϵ is the residual error term. Reference categories were set to fall (season), Friday (weekday), and man (gender).

#### HR Data

HR data were summarized using means and standard deviations for descriptive purposes, stratified by gender and lockdown status. Mixed-effects models were applied to assess differences in HR across time and conditions, with biological gender and lockdown situations as fixed effects (Man and no restrictions were used as reference categories, respectively). The models accounted for the hierarchical structure of the data by including individual-level random effects.

#### Sleep

Total sleep duration was described using means and standard deviations across different conditions. Mixed-effects models evaluated the impact of various variables (fixed effects), including gender (reference: man), PA category (reference: active), year (reference: 2019), lockdown status (reference: no restrictions), and cyclical patterns such as season (reference: fall) and weekday (reference: Friday). The models incorporate individual-level random effects to account for the nested structure of the data.

An alpha risk of .05 was used in statistical tests.

### Ethical Considerations

This study involved a secondary analysis of anonymized data collected via Withings activity trackers. The dataset was fully anonymized at the source, and no identifiable information was accessible to the research team. The study received approval from the internal ethics committee of Institut Curie (approval number DATA210201). During registration for the Withings Health Mate app, all users give consent to the anonymous use of their data for research purposes. They can withdraw this consent at any time and request the deletion of all their data.

Given the anonymization of the dataset, the study complies with applicable privacy regulations, ensuring the confidentiality and protection of participants’ data. No financial compensation was provided to participants, as the data analyzed were part of preexisting anonymized records. In addition, no images or Multimedia Appendices 2 and 3 in this manuscript include identifiable individuals.

## Results

### Characteristics of the Participants

**Figure 1:**
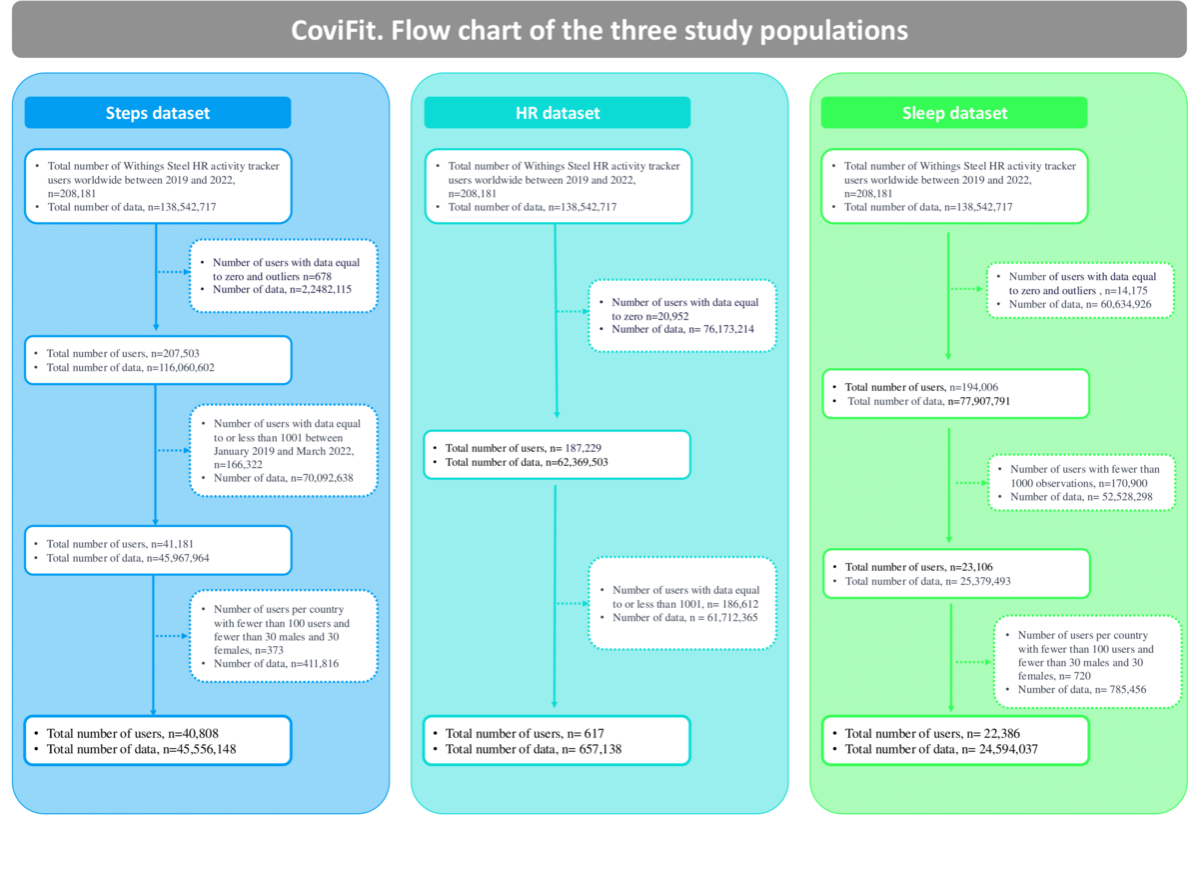
Flowchart of the 3 study populations, detailing the number of users and data points included in each dataset, based on data collected from Withings Steel heart rate activity tracker users across 34 countries.

We collected 138,542,717 data for 208,181 activity tracker users from 34 countries around the world (Figure 1). For the steps dataset, w e analyzed 45,556,148 data points for 40,808 users from 34 countries. The study population was composed of 23,989/40,659 (59%) men and 16,819/41022 (41%) women with a mean age of 47 (SD 12.6) years.

To ensure the robustness of our analysis, various filters were applied to maintain adequate data quality, sufficient monitoring days, and an ample number of individuals within each country’s database. After filtering, we performed a similar analysis on 22,386 users for the sleep dataset and 617 users from the HR dataset (Figure 1). The detailed exploration involved 45,556,148 data points for 40,808 users within the Steps dataset. Similar meticulous data handling was applied to the Sleep and HR datasets, which included 22,386 and 617 users, respectively.

### Changes in the Level of PA (Steps Dataset)

**Figure 2:**
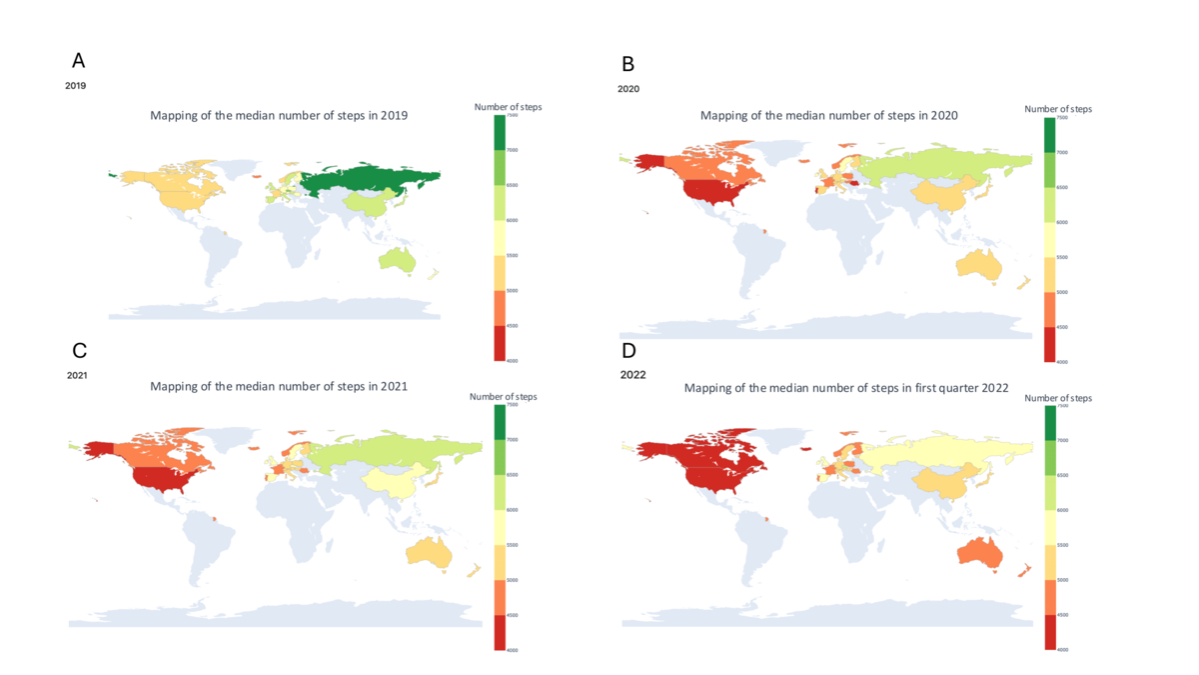
Changes in the median daily step count by country from 2019 to the first quarter of 2022, based on data collected from 40,808 Withings Steel heart rate activity tracker users across 34 countries.

**Figure 3:**
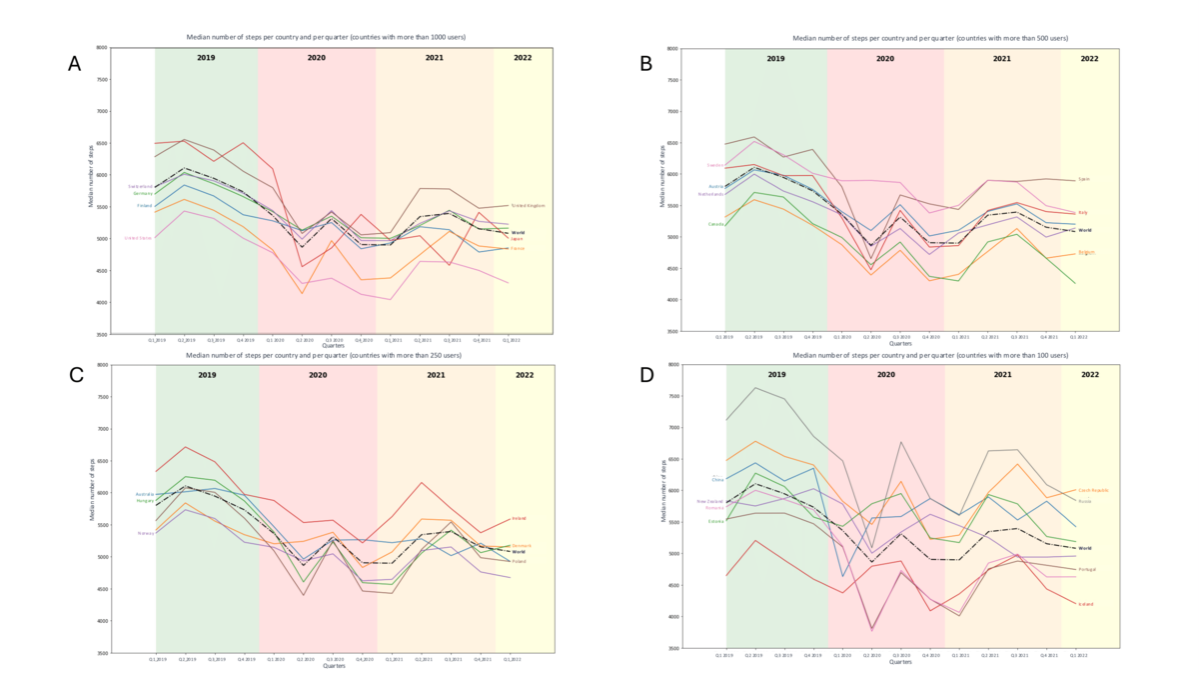
Annual changes in the median number of steps per country, segmented by user base size, illustrate shifts in physical activity among Withings Steel heart rate activity tracker users across 34 countries.

The median number of steps per day in 2019, before the pandemic, was 5700 (IQR 3499-8677) steps /day (Table S1 in Multimedia Appendix 2). Changes in the median daily number of steps taken by individuals per country from 2019 to 2022 are shown in Figures 2 and 3. The color scale in Figure 2 represents the median number of daily steps, ranging from red (fewer steps) to green (more steps), highlighting variations in physical activity levels across countries and over time. Countries with darker green shades indicate higher levels of physical activity, while darker red shades reflect lower activity levels. In Figure 3, changes in the median number of steps taken are displayed across four categories of countries, distinguished by the size of the user base. Category A includes countries with more than 1000 users, Category B with 500 to 999 users, Category C with 250 to 499 users, and Category D with 100 to 249 users. The analysis is visually supported by shaded background colors which represent different years: green for 2019, red for 2020, orange for 2021, and yellow for 2022. This color scheme provides a clear, visual distinction of changes in physical activity levels and user engagement per country over the studied period.

Withings Steel HR activity tracker users were primarily located in Europe (mainly in France, Germany, and the United Kingdom), the United States, and Japan (Figure S1 in Multimedia Appendix 3).

Between 2019 and 2020, there was a significant decrease of 4%-23% in the median number of steps taken daily (Figure 3; Table S1 continued in Multimedia Appendix 2). Between 2019 and 2020, Romania (–23%, from 5821, IQR 3673-8742 to 4472, IQR 2458-7486 steps/d) and Portugal (–20%, from 5572, IQR 3586-8271 to 4469, IQR 2582-7260 steps/d) displayed the largest decreases in the median number of steps taken daily (Figure 3; Figure S1 in Multimedia Appendix 3). Finally, a comparison of the first quarter of 2019 with the first quarter of 2022 revealed a significant decrease in the number of steps worldwide (–12.3%, from 5802 to 5082 steps/d) particularly for Japan (–22.9%, from 6494, IQR 3841-9406 to 5007 IQR 2485-8237 steps/d) and Romania (–19.4%, from 5740, IQR 3631-8565 to 4629, IQR 2642-7540 steps/d), indicating that 2 years after the start of the pandemic, activity levels had not returned to their initial levels, particularly in these countries.

**Figure 4:**
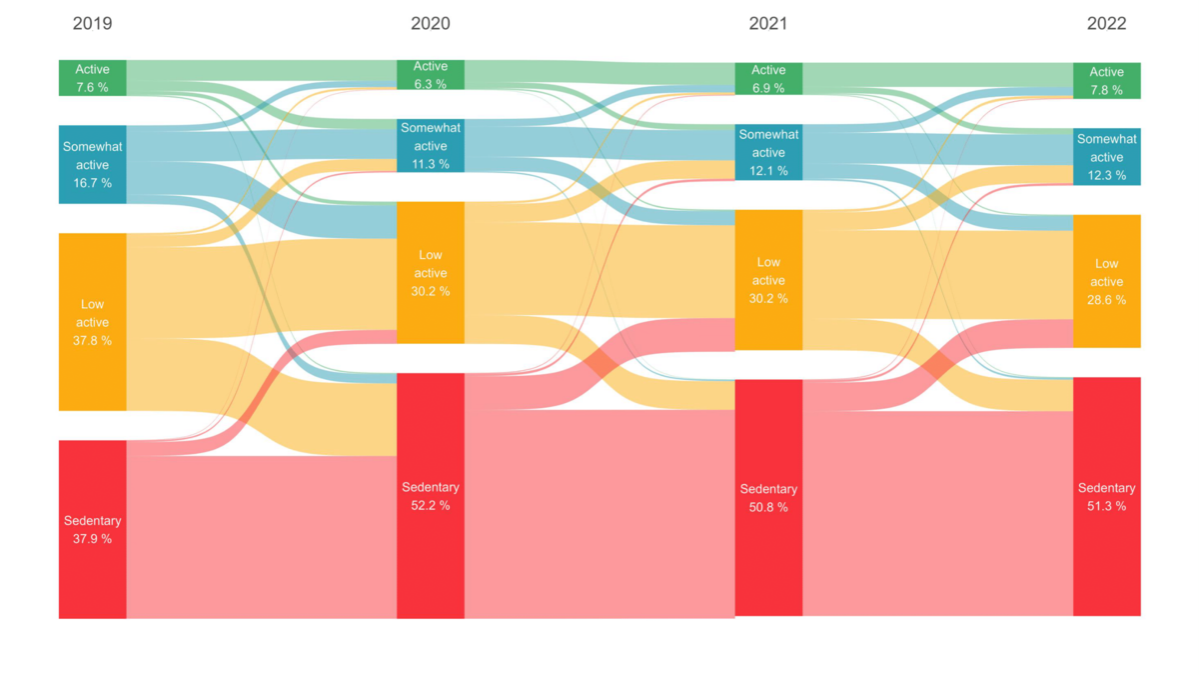
Sankey plot illustrating the distribution of 40,808 Withings Steel heart rate activity tracker across 34 countries within step categories during 2019, 2020, 2021, and the first quarter of 2022.

The proportion of sedentary individuals increased from 38% (n=14,177) in 2019 to 52% (n=19,510) in 2020 and remained elevated at 51% (n=18,972) in 2022, while the proportion of active individuals dropped from 8% (n=2857) to 6% (n=2352) in 2020 before returning to 8% (n=2877) in 2022 (Figure 4). Figure 4 illustrates shifts in activity levels throughout the study period. The various colors within the plot represent different levels of activity among the participants: green indicates active individuals, blue denotes somewhat active individuals, yellow signifies low active individuals, and red represents sedentary individuals. These color-coded flows highlight the transitions between activity categories over time, effectively mapping the changes in physical activity patterns among the study population.

From the mixed-effects models, we estimated: (1) an expected seasonality, with users walking more during the summer (relative to fall as the reference category: α_winter_=–82.3, SE 1.5; α_spring_=191.1, SE 1.6; α_summer_=264.2, SE 1.6), (2) Sunday was the day on which people worldwide walked the least during the week (relative to Friday: α_Saturday_=274.8, SE 2; α_Sunday_=–275.4, SE 2; α_Monday_=–211.1, SE 2; α_Tuesday_=–131.7, SE 2; α_Wednesday_=–128.1, SE 2; α_Thursday_=–100.5, SE 2), and (3) men took more steps daily than women (relative to men, α_women_=–108.3, SE 25.6).

### HR Outcomes

**Figure 5:**
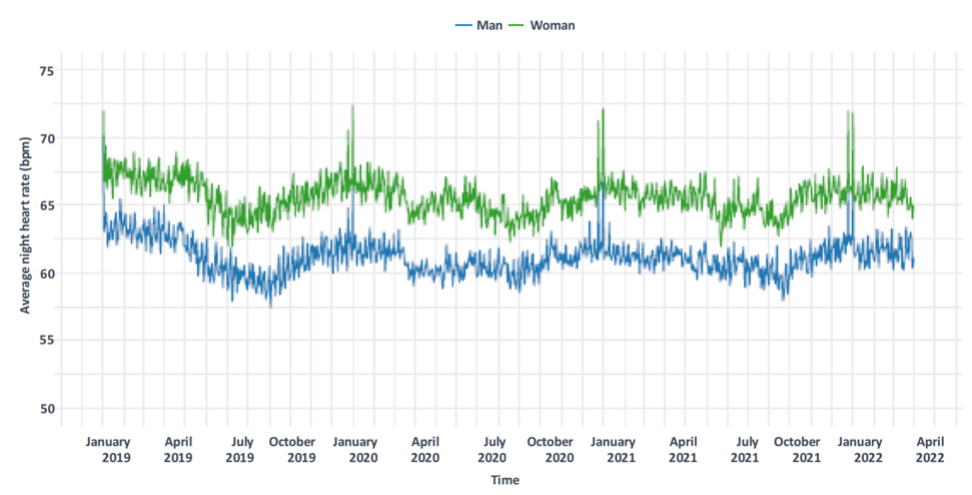
Daily average nighttime heart rate in France from January 2019 to March 2022, based on data from 153 Withings Steel heart rate activity tracker users (95 men and 58 women), with the blue line representing men and the green line representing women.

HR results for France (153 users: 95 men and 58 women) are shown in Figure 5. The mean nighttime HR was 62.8 (SD 8.2) bpm in France. HR was higher in French women than in French men (mean 65.6, SD 7.8 bpm vs mean 61.1, SD 7.9 bpm, respectively) throughout the follow-up period.

Lockdowns were associated with a slight decrease in HR (Figure 5). Mean nighttime HR was usually 62.8 (SD 8.2) bpm in France and 62.5 (SD 8) bpm throughout lockdown periods. Mixed model analysis showed that lockdown situations had a significant impact on resting HR (relative to no restrictions, α_Lockdown_=–0.3±0.1). Several cyclic patterns were also observed, with seasonal variations (HR higher in winter than in summer) and peaks related to Christmas or New Year celebrations (Figure 5).

### Sleep

The average total sleep duration for all countries was 7.5 (SD 1.4) hours. On an average, men slept for 19.5 (SD 0.6) minutes less than women. Active users also slept less than sedentary users (compared with active users: α_somewhat active_=4.1, SE 1.2; α_low active_=9.3, SE 1.1; α_sedentary_=SD, SE 1.1). In 2019, mean sleep duration was 15 minutes shorter than in the other years (relative to 2019: α_2020_=15.7, SE 0; α_2021_=15.1, SE 0’ α_2022_=17, SE 0.1). Sleep duration increased by 4.8±0.1 minutes in countries implementing strict lockdowns (eg, France, Italy, and Spain).

Several cyclic patterns were observed in the variation of sleep duration. Over a week, sleep duration was 30 minutes longer during weekends (relative to Friday: α_Saturday_=25.9, SE 0.1; α_Sunday_=32.6, SE 0.1; α_Monday_=0.8, SE 0.1; α_Tuesday_=–0.8, SE 0.1; α_Wednesday_=–0.4, SE 0.1; α_Thursday_=–0.4, SE 0.1). Over a year, sleep duration was longer in winter than in the fall, and significantly shorter in spring and summer (relative to fall: α_winter_=3.7, SE 0; α_spring_=–3.1 SD 0; α_summer_=–6.5, SE 0).

## Discussion

### Principal Findings

Our study highlights a persistent decline in PA levels worldwide, with a significant 12.3% (from 5802 to 5082 steps/d) decrease in average step count from the first quarter of 2019 to the first quarter of 2022, even among active users. This decline persisted regardless of the stringency of the measures taken by the government or the severity of the pandemic in the country concerned. The pandemic, thus, had an impact on lifestyle behaviors that persisted 2 years later. In addition, the proportion of individuals with very low levels of PA increased considerably, from 38% (n=14,177) in 2019 to 52% (n=19,510) in 2020.

### PA Changes

#### PA Decline

Our results indicate a less significant decrease in PA levels than reported in other studies conducted in different countries [3]. A British cohort study demonstrated a 30% decrease in PA levels, whereas another study comparing Swiss and Brazilian participants found a 23.7% overall decrease in PA levels, with 31.6% of Brazilians and 15.8% of Swiss individuals displaying lower levels of PA [34]. Furthermore, the impact of the pandemic on PA depended on prepandemic activity levels. In our study, the most affected groups in 2020 were those who were moderately or highly active before the restrictions were imposed. This finding is consistent with the results of a survey conducted across 14 countries that showed a 41% decrease in moderate-intensity activities and a 42% decrease in vigorous activities [5]. However, the decline was particularly pronounced among those who were already less active, as they struggled to regain their prepandemic activity levels, making this group the most vulnerable and at higher risk of chronic diseases. Building on this, emerging global data indicate that PA levels have continued to decline significantly even after the pandemic. A recent analysis indicated that nearly 1.8 billion adults worldwide did not meet recommended PA levels in 2022, reflecting a 5% increase in global inactivity since 2010 [35]. The WHO 2024 Global Status Report on Physical Activity corroborates this trend, projecting that 35% of the global adult population could be physically inactive by 2030 [36]. Factors such as pandemic-induced behavioral changes, prolonged sedentary habits, and socioeconomic barriers have likely contributed to these persistent declines. These findings underscore the pressing need for targeted interventions to promote PA and address the structural challenges that hinder its recovery.

#### Age and Sex Inequalities

Previous studies have indicated a greater decline in PA among young adults compared to older individuals [37-39], whereas 2 other studies reported the opposite trend [40,41]. However, many studies showed that the individuals most affected by the pandemic in terms of PA levels were those with preexisting health conditions, and those from lower socioeconomic groups [5,34]. These populations often face significant challenges in maintaining their levels of PA due to higher health risks, limited access to exercise facilities, and exacerbated social inequalities during the pandemic. In our study, PA declined and sedentary behaviors increased in both sexes, with a stronger impact observed in women, as previously reported [16].

#### Seasonality

We found that both day of the week and season influenced PA levels with participants being least active on Sundays and in winter [42,43]. These results are consistent with other studies showing that PA levels typically decrease during winter due to colder weather, reduced daylight, and increased indoor activities, while higher levels are observed in summer [44,45]. The alignment of our findings with established patterns in PA seasonality supports the representativeness and robustness of our data, as similar trends have been observed across various countries and populations.

Prepandemic studies consistently demonstrated higher PA levels in spring and summer compared with fall and winter, driven by environmental factors such as increased daylight hours and favorable weather conditions [44,46]. During the pandemic, these seasonal trends persisted but were further amplified by external stressors, such as lockdowns and mobility restrictions, particularly during the winter months when outdoor activities were further limited. Postpandemic data also reflect these same seasonality patterns, indicating that while the pandemic introduced new behavioral stressors, it did not fundamentally alter the established influence of seasonality on PA levels.

This seasonal variation in PA should be considered when interpreting the results and planning public health interventions aimed at increasing activity levels year-round.

### Change in Heart Rate

Our results are consistent with previous findings indicating that women generally have a higher resting HR than men (mean difference of 3-5 bpm), primarily due to physiological differences in heart size, hormonal influences, and autonomic regulation [47]. Regular PA is known to lower resting HR by improving parasympathetic tone [48-50]. The COVID-19 pandemic led to significant lifestyle changes, including a decrease in PA and increased stress, both of which can affect resting HR and its variability. During infections, including COVID-19, HR tends to rise due to immune and inflammatory responses, as well as increased metabolic rate and sympathetic nervous system activation [51]. HR data from activity trackers provide a potentially valuable metric for monitoring, identifying at-risk individuals, and assessing behavioral changes. For instance, our data indicate a similar one-night increase in HR across the 34 countries studied on December 31, probably due to late-night partying and alcohol consumption [52,53].

### Changes to Sleep Patterns

In this study, we observed a general increase in sleep duration during the pandemic, likely driven by more flexible work schedules and reduced commuting times. However, these findings must be interpreted within the broader context of sleep research. Several studies have shown that insufficient sleep is associated with adverse health outcomes, including an increased risk of chronic conditions such as obesity, diabetes, cardiovascular disease, and mental health disorders [54-56].

Beyond sleep duration, researchers have emphasized the importance of “sleep health,” which encompasses sleep efficiency, disturbances, and time spent in various sleep phases [57,58]. Our analysis aligns with studies documenting an increase in sleep duration during the pandemic [59-61], reflecting reduced social and commuting demands, as well as seasonal and weekend variations, with longer sleep durations observed in winter and weekends [62]. However, the pandemic has also been associated with deteriorations in sleep quality and disrupted sleep-wake cycles, highlighting the complex interplay between behavioral and environmental factors. Furthermore, the sex-specific differences in sleep patterns observed in this study are consistent with previous findings, emphasizing the importance of considering these differences in future analyses [63]. Understanding these patterns is essential for developing interventions to improve sleep health during disruptive events such as a pandemic. Future research should explore how sleep behaviors adapt to such stressors over time, integrating objective measures of sleep quality alongside sleep duration for a more comprehensive understanding of sleep health.

### Public Health Implications

One study assessed the impact of pandemic-related measures on step count declines and their correlation with the emergence of chronic diseases and increases in overall mortality [17,64]. This study, based on data collected from activity trackers in 60 countries between January 2020 and January 2022, showed that greater restriction severity was associated with lower step counts and a nonlinear increase in the modeled risk of all-cause mortality, by up to 40% [64]. This finding underscores the significant health risks linked to reduced PA during extended lockdowns, such as those experienced in Spain or France [3].

The ongoing global decline in PA remains a major public health issue, as low levels of activity have been linked to higher risks of chronic diseases, such as cancer, heart disease, and diabetes [14,17,65]. The pandemic not only exacerbated physical inactivity but also contributed to an average weight gain of 2 kg [66]. Furthermore, the long-term health implications of these behavioral changes extend beyond PA. Sustained inactivity and prolonged sedentary behavior have been associated with metabolic dysfunction, reduced cardiovascular fitness, and an elevated risk of mental health disorders [67]. Similarly, while the increase in sleep duration observed during the pandemic may offer short-term benefits, persistent disruptions in sleep quality, and circadian rhythms could negatively affect cognitive functioning, metabolic health, and overall well-being [68]. Addressing these risks requires targeted interventions that go beyond restoring prepandemic activity and sleep levels to promote sustainable, healthy behaviors.

Long-standing habits of physical inactivity are notoriously difficult to change, as they are deeply ingrained in daily routines and influenced by multiple environmental and social factors. Research suggests that years of sustained effort are needed to increase PA levels across populations, and setbacks, such as pandemic-related lockdowns, can rapidly reverse these gains [69]. These findings highlight the urgent need for efficient strategies to enhance PA at multiple levels: individual, community, regional, national, and continental. Such strategies are essential to mitigate the adverse health impacts of increasing sedentary behavior.

Data collected through wearable devices demonstrate the potential for personalized feedback to drive behavior change. These devices can deliver actionable insights, enabling users to monitor and adjust their PA, heart rate, and sleep patterns in real time. Research demonstrates that real-time feedback mechanisms, such as notifications, goal-setting, and tailored trend analyses, can significantly improve user engagement and adherence to PA recommendations [28,70]. Furthermore, such devices can identify behaviors indicative of potential health risks, prompting timely interventions. Public health strategies should explore the integration of wearable technologies into intervention programs, leveraging these devices to empower individuals and foster healthier lifestyle choices.

### Study Strengths and Limitations

The use of digital health technologies, such as activity trackers, has emerged as a key strategy for monitoring and potentially improving health metrics. These tools provide users with real-time feedback, which can be instrumental in promoting PA [23-29]. The data collected through such devices in our study offer valuable insight into the impact of the pandemic on PA, HR, and sleep patterns, providing a model for the use of digital tools in public health surveillance and interventions. Integrating these findings into user-friendly platforms with actionable insight could further enhance the efficacy of these tools for promoting health and well-being.

This study highlights trends in PA among activity tracker users during the COVID-19 pandemic. A major strength is the extensive collection of real-life data from over 200,000 users across 34 countries over a 3-year period including a full baseline year (2019) before the pandemic. The detailed data granularity, large sample size, and inclusion of sociodemographic and anthropometric information enable accurate population characterization.

However, this study has several limitations. First, the population of Withings Steel HR activity tracker users may not fully represent the general population, particularly in countries with low numbers of users. For example, wearable technology users tend to be more health-conscious, potentially introducing selection bias [71]. Nonetheless, the weighted average BMI across countries with more than 1000 users (26.35 kg/m²) aligns with averages for Europe and North America and is only slightly above the global average (24.9 kg/m²) [72]. Similarly, the weighted median daily step count (5952 steps/d) corresponds closely to global trends reported in activity tracker studies [12]. While our sample may include more health-conscious individuals, it is representative of broader population activity trends. The absence of socioeconomic data, however, limits a comprehensive assessment of representativeness.

Although age was included as a covariate to assess its impact on PA, heart rate, and sleep patterns, we could not control for preexisting health conditions due to data limitations. This may have introduced residual confounding, as individuals with chronic health conditions often exhibit distinct behavioral and physiological responses to the pandemic.

Second, the study did not account for differences in the severity of the pandemic across regions. COVID-19 severity and associated public health measures varied significantly not only between countries but also within countries. For instance, in the United States, individual states implemented different lockdown measures, while in Germany, the Länder adopted diverse approaches to pandemic management [73,74]. These variations likely influenced public behavior, including PA and mobility patterns [75]. However, our dataset only included country-level information, precluding the analysis of regional heterogeneity. Future research incorporating regional data could provide more granular insights.

Third, the influence of seasonality on PA distribution complicates cross-country comparisons. In addition, the analysis for 2022 only includes data from the first quarter, potentially introducing seasonal bias, as PA levels are generally lower in winter. Fourth, measuring PA based solely on step counts may underestimate total activity, particularly in cases of increased cycling, swimming, or strength training during the pandemic.

Future studies should incorporate additional metrics, such as accelerometer-based wearables or self-reported activity logs, to capture the full spectrum of PA behaviors. Developing algorithms to assess activity levels and sedentary durations across different activities could also improve comparability.

### Conclusions

The COVID-19 pandemic has significantly decreased global PA levels, especially among inactive or sedentary individuals. Our study of data for more than 200,000 activity tracker users in 34 countries showed a 12.3% reduction in daily step count from 2019 to 2022. A 1.5% decrease in HR occurred during lockdowns, associated with reduced activity levels. Sleep duration increased during restrictions, particularly in the countries with strict lockdown measures. These findings underscore the urgent need for public health strategies to promote active lifestyles, counteract sedentary behaviors, and mitigate long-term health risks. Furthermore, our results highlight the potential of wearable devices not only for monitoring health metrics but also for facilitating real-time feedback to support behavior change. Promoting PA as a key component of global health resilience is essential during and after disruptive events such as pandemics. Future public health initiatives should integrate technology-based interventions to encourage healthier lifestyles and strengthen population-level health outcomes.

## Appendix

Multimedia Appendix 1 STROBE (Strengthening the Reporting of Observational Studies in Epidemiology) checklist.

Multimedia Appendix 2 Baseline demographic, anthropometric and physical activity characteristics of the study population in 2019, based on data collected from Withings Steel HR activity tracker users in 34 countries.

Multimedia Appendix 3 Variation in the number of steps per quarter.

## Abbreviations

HR: heart rate
PA: physical activity
STROBE: Strengthening the Reporting of Observational Studies in Epidemiology
WHO: World Health Organization

## Data Availability

The datasets generated or analyzed during this study are not publicly available due to the volume of data and the personal nature of the data but are available from the corresponding author on reasonable request.
