# Supplementary material for: Changes in Physical Activity, Heart Rate, and Sleep Measured by Activity Trackers During the COVID-19 Pandemic Across 34 Countries: Retrospective Analysis
Source: J Med Internet Res. 2025 Apr 4;27:e68199. doi: 10.2196/68199 (PMC12008701; doi:10.2196/68199)
Supplement: Multimedia Appendix 2 [file jmir_v27i1e68199_app2.pdf]

**Table 1.** Baseline demographic, anthropometric and physical activity characteristics of the study population in 2019, based on data collected from Withings Steel HR activity tracker users in 34 countries.

| **Baseline characteristics (in 2019)** | **Australia (*n*=461)** | **Austria (*n*=554)** | **Belgium (*n*=847)** | **Canada (*n*=947)** | **China (*n*=167)** | **Czech Republic (*n*=196)** | **Denmark (*n*=413)** | **Estonia (*n*=151)** | **Finland (*n*=1,196)** | **France (*n*=9,800)** | **Germany (*n*=7,565)** |
|---|---|---|---|---|---|---|---|---|---|---|---|
| **Sex** (*n*, %) | | | | | | | | | | | |
| Women | 221 (47.94) | 243 (43.86) | 380 (44.86) | 371 (39.18) | 29 (17.37) | 67 (34.18) | 178 (43.1) | 79 (52.32) | 460 (38.46) | 4495 (45.87) | 3281 (43.37) |
| Men | 240 (52.06) | 311 (56.14) | 467 (55.14) | 576 (60.82) | 138 (82.63) | 129 (65.82) | 235 (56.9) | 72 (47.68) | 736 (61.54) | 5305 (54.13) | 4284 (56.63) |
| **Age (years)** (mean, sd) | 48 (13.36) | 50 (13.51) | 47 (12.84) | 49 (13.96) | 39 (10.08) | 41 (12.1) | 49 (13.6) | 42 (9.73) | 48 (12.82) | 49 (13.53) | 51 (12.81) |
| **Age categories (years)** (*n*, %) | | | | | | | | | | | |
| 18-24 | 7 (1.52) | 10 (1.81) | 16 (1.89) | 14 (1.48) | 10 (5.99) | 9 (4.59) | 9 (2.18) | 4 (2.65) | 32 (2.68) | 168 (1.71) | 124 (1.64) |
| 25-39 | 141 (30.59) | 146 (26.35) | 277 (32.70) | 296 (31.26) | 94 (56.29) | 103 (52.55) | 115 (27.85) | 68 (45.03) | 333 (27.84) | 2980 (30.41) | 1632 (21.57) |
| 40-54 | 159 (34.49) | 197 (35.56) | 341 (40.26) | 325 (34.32) | 52 (31.14) | 54 (27.55) | 167 (40.44) | 63 (41.72) | 502 (41.97) | 3620 (36.94) | 2798 (36.99) |
| 55-64 | 105 (22.78) | 122 (22.02) | 144 (17.00) | 177 (18.69) | 7 (4.19) | 21 (10.71) | 76 (18.40) | 12 (7.95) | 196 (16.39) | 1795 (18.32) | 2046 (27.05) |
| ≥ 65 | 49 (10.63) | 79 (14.26) | 68 (8.03) | 135 (14.26) | 4 (2.40) | 4 (2.04 ) | 46 (11.14) | 4 (2.65) | 133 (11.12) | 1234 (12.59) | 965 (12.76) |
| **BMI** (mean, sd) | 27 (5.09) | 26 (4.93) | 26 (4.33) | 27 (4.86) | 24 (3.29) | 26 (4.73) | 26 (4.49) | 26 (4.94) | 27 (4.67) | 26 (4.51) | 27 (4.75) |
| **BMI categories** (*n*,%) | | | | | | | | | | | |
| Underweight (<18.5 kg/m$^2$) | 4 (0.87) | 4 (0.72) | 7 (0.83) | 6 (0.63) | 4 (2.4) | 3 (1.53) | 2 (0.48) | 3 (1.99) | 7 (0.59) | 143 (1.46) | 61 (0.81) |
| Normal weight (<25 kg/m$^2$) | 226 (49.02) | 282 (50.9) | 431 (50.89) | 430 (45.41) | 118 (70.66) | 87 (44.39) | 195 (47.22) | 72 (47.68) | 557 (46.57) | 5194 (53.0) | 3417 (45.17) |
| Overweight (25-30 kg/m$^2$) | 4 (0.87) | 4 (0.72) | 7 (0.83) | 6 (0.63) | 4 (2.4) | 3 (1.53) | 2 (0.48) | 3 (1.99) | 7 (0.59) | 143 (1.46) | 2720 (35.96) |

| | | | | | | | | | | | |
|---|---|---|---|---|---|---|---|---|---|---|---|
| Obese (>30 kg/m2) | 79 (17.14) | 88 (15.88) | 115 (13.58) | 153 (16.16) | 4 (2.4) | 30 (15.31) | 66 (15.98) | 27 (17.88) | 235 (19.65) | 1314 (13.41) | 1367 (18.07) |
| **Number of steps** (median) | | | | | | | | | | | |
| Sedentary | 152 (32.97) | 193 (34.84) | 365 (43.09) | 394 (41.61) | 42 (25.15) | 47 (23.98) | 161 (38.98) | 55 (36.42) | 452 (37.79) | 4169 (42.54) | 2718 (35.93) |
| Low active | 173 (37.53) | 229 (41.34) | 331 (39.08) | 354 (37.38) | 81 (48.5) | 83 (42.35) | 179 (43.34) | 58 (38.41) | 521 (43.56) | 3611 (36.85) | 2958 (39.1) |
| Somewhat active | 96 (20.82) | 84 (15.16) | 111 (13.11) | 137 (14.47) | 30 (17.96) | 41 (20.92) | 49 (11.86) | 23 (15.23) | 158 (13.21) | 1420 (14.49) | 1276 (16.87) |
| Active | 40 (8.68) | 48 (8.66) | 40 (4.72) | 62 (6.55) | 14 (8.38) | 25 (12.76) | 24 (5.81) | 15 (9.93) | 65 (5.43) | 600 (6.12) | 613 (8.1) |

**Table 1** Continued. Baseline demographic, anthropometric and physical activity characteristics of the study population in 2019, based on data collected from Withings Steel HR activity tracker users in 34 countries.

| **Baseline characteristics (in 2019)** | **Hong Kong (*n*=79)** | **Hungary (*n*=268)** | **Iceland (*n*=109)** | **India (*n*=56)** | **Ireland (*n*=252)** | **Italy (*n*=719)** | **Japan (*n*=1 948)** | **Mexico (*n*=67)** | **Nether lands (*n*=854)** | **New Zealand (*n*=143)** | **Norway (*n*=339)** |
|---|---|---|---|---|---|---|---|---|---|---|---|
| **Sex** (*n*, %) | | | | | | | | | | | |
| Women | 17 (21.52) | 71 (26.49) | 63 (57.8) | 9 (16.07) | 100 (39.68) | 202 (28.09) | 402 (20.64) | 18 (26.87) | 382 (44.73) | 76 (53.15) | 145 (42.77) |
| Men | 62 (78.48) | 197 (73.51) | 46 (42.2) | 47 (83.93) | 152 (60.32) | 517 (71.91) | 1546 (79.36) | 49 (73.13) | 472 (55.27) | 67 (46.85) | 194 (57.23) |
| **Age (years)** (mean, sd) | 46 (11.05) | 43 (10.98) | 49 (11.83) | 46 (13.35) | 47 (12.81) | 49 (12.6) | 48 (11.13) | 49 (14.73) | 48 (13.22) | 46 (13.32) | 47 (13.13) |
| **Age categories (years)** (*n*, %) | | | | | | | | | | | |
| 18-24 | 1 (1.27) | 12 (4.48) | 1 (0.92) | 1 (1.79) | 3 (1.19) | 9 (1.25) | 12 (0.61) | 2 (2.99) | 21 (2.46) | 3 (2.10) | 5 (1.47) |
| 25-39 | 28 (35.44) | 99 (36.94) | 26 (23.85) | 22 (39.29) | 82 (32.54) | 193 (26.84) | 517 (26.54) | 19 (28.36) | 246 (28.81) | 55 (38.46) | 127 (37.46) |
| 40-54 | 32 (40.51) | 127 (47.39) | 46 (42.20) | 19 (33.93) | 98 (38.89) | 311 (43.25) | 910 (46.71) | 24 (35.82) | 310 (36.30) | 46 (32.17) | 113 (33.33) |
| 55-64 | 15 (18.99) | 18 (6.72) | 28 (25.69) | 11 (19.64) | 47 (18.65) | 130 (18.08) | 378 (19.40) | 13 (19.40) | 184 (21.55) | 28 (19.58) | 65 (19.17) |
| ≥ 65 | 3 (3.80) | 12 (4.48) | 8 (7.34) | 3 (5.36) | 21 (8.33) | 76 (10.57) | 127 (6.52) | 9 (13.43) | 89 (10.42) | 10 (6.99) | 28 (8.26) |
| **BMI** (mean, sd) | 24 (3.95) | 26 (4.02) | 28 (5.2) | 27 (5.01) | 27 (4.61) | 25 (4.0) | 24 (3.66) | 27 (4.56) | 26 (4.51) | 27 (4.69) | 26 (4.49) |
| **BMI categories** (*n*,%) | | | | | | | | | | | |
| Underweight (<18.5 kg/m$^2$) | 2 (2.53) | 2 (0.75) | 0 (0.0) | 1 (1.79) | 2 (0.79) | 10 (1.39) | 59 (3.03) | 0 (0.0) | 13 (1.52) | 0 (0.0) | 3 (0.88) |

| | | | | | | | | | | | |
|---|---|---|---|---|---|---|---|---|---|---|---|
| Normal weight (<25 kg/m$^2$) | 55 (69.62) | 139 (51.87) | 34 (31.19) | 25 (44.64) | 105 (41.67) | 404 (56.19) | 1325 (68.02) | 24 (35.82) | 432 (50.59) | 60 (41.96) | 174 (51.33) |
| Overweight (25-30 kg/m$^2$) | 15 (18.99) | 95 (35.45) | 41 (37.61) | 22 (39.29) | 96 (38.1) | 236 (32.82) | 454 (23.31) | 34 (50.75) | 297 (34.78) | 52 (36.36) | 119 (35.1) |
| Obese (>30 kg/m$^{2)}$ | 7 (8.86) | 32 (11.94) | 34 (31.19) | 8 (14.29) | 49 (19.44) | 69 (9.6) | 110 (5.65) | 9 (13.43) | 112 (13.11) | 31 (21.68) | 43 (12.68) |
| **Number of steps** (median) | | | | | | | | | | | |
| Sedentary | 10 (12.66) | 81 (30.22) | 59 (54.13) | 26 (46.43) | 71 (28.17) | 227 (31.57) | 530 (27.21) | 28 (41.79) | 307 (35.95) | 46 (32.17) | 132 (38.94) |
| Low active | 31 (39.24) | 112 (41.79) | 37 (33.94) | 20 (35.71) | 95 (37.7) | 281 (39.08) | 716 (36.76) | 25 (37.31) | 358 (41.92) | 58 (40.56) | 142 (41.89) |
| Somewhat active | 27 (34.18) | 52 (19.4) | 9 (8.26) | 8 (14.29) | 61 (24.21) | 155 (21.56) | 493 (25.31) | 12 (17.91) | 137 (16.04) | 26 (18.18) | 53 (15.63) |
| Active | 11 (13.92) | 23 (8.58) | 4 (3.67) | 2 (3.57) | 25 (9.92) | 56 (7.79) | 209 (10.73) | 2 (2.99) | 52 (6.09) | 13 (9.09) | 12 (3.54) |

**Table 1 Continued**. Baseline demographic, anthropometric and physical activity characteristics of the study population in 2019, based on data collected from Withings Steel HR activity tracker users in 34 countries.

| **Baseline characteristics (in 2019)** | **Poland (*n*=331)** | **Portugal (*n*=245)** | **Romania (*n*=205)** | **Russia (*n*=144)** | **Singapore (*n*=73)** | **Spain (*n*=672)** | **Sweden (*n*=653)** | **Switzer land (*n*=1674)** | **Thailand (*n*=77)** | **United Kingdom (*n*=4057)** | **United States (*n*=5898)** | **Vietn am (*n*=2 1)** |
|---|---|---|---|---|---|---|---|---|---|---|---|---|
| **Sex** (*n*, %) | | | | | | | | | | | | |
| Women | 98 (29.61) | 85 (34.69) | 77 (37.56) | 47 (32.64) | 26 (35.62) | 237 (35.27) | 245 (37.52) | 818 (48.86) | 18 (23.38) | 1635 (40.3) | 2332 (39.54) | 4 (19.05) |
| Men | 233 (70.39) | 160 (65.31) | 128 (62.44) | 97 (67.36) | 47 (64.38) | 435 (64.73) | 408 (62.48) | 856 (51.14) | 59 (76.62) | 2422 (59.7) | 3566 (60.46) | 17 (80.95) |
| **Age (years)** (mean, sd) | 43 (10.29) | 49 (13.48) | 43 (11.38) | 47 (12.42) | 46 (10.66) | 50 (13.19) | 49 (12.69) | 49 (13.66) | 43 (11.37) | 50 (13.29) | 49 (13.78) | 43 (8.58) |
| **Age (years) categories** (*n*, %) | | | | | | | | | | | | |
| 18-24 | 4 | 1 | 3 | 3 | 2 | 11 | 11 | 33 | 2 | 63 | 79 | 0 |
| 25-39 | 143 | 74 | 93 | 45 | 20 | 163 | 176 | 480 | 30 | 1053 | 1880 | 7 |
| 40-54 | 143 | 93 | 79 | 63 | 37 | 278 | 262 | 591 | 33 | 1501 | 1915 | 12 |
| 55-64 | 31 | 38 | 17 | 21 | 10 | 125 | 131 | 356 | 9 | 891 | 1190 | 2 |
| ≥ 65 | 10 | 39 | 12 | 12 | 4 | 95 | 69 | 213 | 1 | 549 | 834 | 23 (3.44) |
| **BMI** (mean, sd) | 26 (4.5) | 26 (4.04) | 26 (4.35) | 26 (4.55) | 25 (4.13) | 26 (4.15) | 26 (4.69) | 26 (4.61) | 24 (3.91) | 27 (4.76) | 27 (5.16) | 2 (9.52) |
| **BMI categories** (*n*,%) | | | | | | | | | | | | |
| Underweight (<18.5 kg/m$^2$) | 7 (2.11) | 3 (1.22) | 1 (0.49) | 1 (0.69) | 2 (2.74) | 8 (1.19) | 7 (1.07) | 20 (1.19) | 3 (3.9) | 34 (0.84) | 55 (0.93) | 14 (66.67) |
| Normal weight (<25 kg/m$^2$) | 163 (49.24) | 133 (54.29) | 104 (50.73) | 70 (48.61) | 45 (61.64) | 361 (53.72) | 312 (47.78) | 902 (53.88) | 57 (74.03) | 1897 (46.76) | 2490 (42.22) | 5 (23.81) |

| | | | | | | | | | | | | |
|---|---|---|---|---|---|---|---|---|---|---|---|---|
| Overweight (25-30 kg/m$^2$) | 117 (35.35) | 84 (34.29) | 75 (36.59) | 53 (36.81) | 21 (28.77) | 231 (34.38) | 217 (33.23) | 516 (30.82) | 10 (12.99) | 1431 (35.27) | 2103 (35.66) | 0 (0.0) |
| Obese (>30 kg/m$^{2)}$ | 44 (13.29) | 25 (10.2) | 25 (12.2) | 20 (13.89) | 5 (6.85) | 72 (10.71) | 117 (17.92) | 236 (14.1) | 7 (9.09) | 695 (17.13) | 1250 (21.19) | 8 (38.1) |
| **Number of steps** (median) | | | | | | | | | | | | |
| Sedentary | 113 (34.14) | 90 (36.73) | 67 (32.68) | 28 (19.44) | 19 (26.03) | 174 (25.89) | 183 (28.02) | 581 (34.71) | 35 (45.45) | 1203 (29.65) | 2684 (45.51) | 7 (33.33) |
| Low active | 151 (45.62) | 108 (44.08) | 90 (43.9) | 48 (33.33) | 37 (50.68) | 273 (40.62) | 279 (42.73) | 687 (41.04) | 32 (41.56) | 1468 (36.18) | 2051 (34.77) | 3 (14.29) |
| Somewhat active | 43 (12.99) | 32 (13.06) | 32 (15.61) | 37 (25.69) | 14 (19.18) | 142 (21.13) | 124 (18.99) | 273 (16.31) | 8 (10.39) | 922 (22.73) | 799 (13.55) | 3 (14.29) |
| Active | 24 (7.25) | 15 (6.12) | 16 (7.8) | 31 (21.53) | 3 (4.11) | 83 (12.35) | 67 (10.26) | 133 (7.95) | 2 (2.6) | 464 (11.44) | 364 (6.17) | NaN |
