# Supplementary material for: Changes in Physical Activity, Heart Rate, and Sleep Measured by Activity Trackers During the COVID-19 Pandemic Across 34 Countries: Retrospective Analysis
Source: J Med Internet Res. 2025 Apr 4;27:e68199. doi: 10.2196/68199 (PMC12008701; doi:10.2196/68199)
Supplement: Multimedia Appendix 3 [file jmir_v27i1e68199_app3.pdf]

		1st Quarter			2nd Quarter		3rd Quarter		4th Quarter	
**Country**	**n=**	**Var Q1_20/Q1_19**	**Var Q1_21/Q1_19**	**Var Q1_22/Q1_19**	**Var Q2_20/Q2_19**	**Var Q2_21/Q2_19**	**Var Q3_20/Q3_19**	**Var Q3_21/Q3_19**	**Var Q4_20/Q3_19**	**Var Q4_21/Q4_19**
Australia	461	-8,4%	-12,6%	-17,5%	-17,5%	-12,2%	-13,3%	-17,3%	-11,6%	-12,6%
Austria	554	-6,4%	-11,5%	-9,8%	-15,9%	-10,9%	-7,8%	-7,6%	-12,9%	-9,2%
Belgium	847	-8,3%	-17,2%	-11,1%	-21,4%	-14,8%	-12,0%	-5,7%	-17,1%	-10,1%
Canada	947	-3,5%	-17,0%	-17,7%	-20,2%	-13,8%	-12,7%	-10,5%	-16,1%	-10,5%
China	167	-25,1%	-9,2%	-12,3%	-13,6%	-8,4%	-9,2%	-10,1%	-7,6%	-8,2%
Czech Republic	196	-10,0%	-18,3%	-7,2%	-19,5%	-12,0%	-6,1%	-1,9%	-18,4%	-8,1%
Denmark	413	-4,0%	-6,3%	-4,9%	-10,3%	-4,3%	-3,1%	0,2%	-9,6%	-3,3%
Estonia	151	-1,6%	-6,3%	-6,0%	-7,7%	-5,4%	-1,8%	-4,6%	-5,9%	-5,5%
Finland	1196	-4,1%	-10,4%	-11,8%	-12,2%	-11,2%	-7,4%	-9,4%	-10,0%	-10,9%
France	9800	-10,9%	-19,0%	-10,7%	-26,3%	-15,4%	-8,7%	-6,1%	-16,0%	-5,8%
Germany	7565	-4,8%	-12,2%	-9,4%	-15,0%	-13,8%	-8,7%	-7,1%	-11,4%	-9,0%
Hungary	268	-8,4%	-22,3%	-11,9%	-26,3%	-19,1%	-15,6%	-12,6%	-21,7%	-13,7%
Iceland	109	-5,9%	-6,3%	-9,6%	-7,8%	-9,0%	-0,3%	1,7%	-10,9%	-3,4%
Ireland	252	-7,2%	-11,1%	-11,7%	-17,6%	-8,3%	-14,1%	-11,3%	-12,7%	-10,0%
Italy	719	-13,0%	-20,2%	-12,0%	-27,2%	-11,9%	-9,2%	-7,1%	-19,0%	-9,6%
Japan	1948	-6,2%	-23,5%	-22,9%	-30,2%	-22,7%	-21,9%	-26,3%	-17,3%	-16,8%
Netherlands	854	-5,7%	-10,8%	-9,4%	-19,1%	-13,5%	-10,4%	-7,2%	-15,1%	-10,1%
New Zealand	143	-0,9%	-6,7%	-15,0%	-13,0%	-8,7%	-9,1%	-15,8%	-6,7%	-18,0%
Norway	339	-4,2%	-13,4%	-12,9%	-13,9%	-11,1%	-9,8%	-7,9%	-11,5%	-8,9%
Poland	331	-8,5%	-20,4%	-11,5%	-27,6%	-15,8%	-12,5%	-7,7%	-20,3%	-11,0%
Portugal	245	-8,0%	-27,7%	-14,4%	-32,4%	-15,7%	-16,7%	-13,5%	-21,8%	-12,0%
Romania	205	-10,9%	-29,2%	-19,4%	-37,3%	-19,2%	-19,2%	-14,8%	-24,9%	-18,7%
Russia	144	-9,1%	-21,3%	-17,9%	-33,3%	-13,1%	-9,1%	-10,8%	-14,4%	-11,2%
Spain	672	-10,5%	-16,1%	-9,0%	-29,4%	-10,5%	-9,6%	-6,2%	-13,6%	-7,3%
Sweden	653	-4,1%	-10,4%	-12,3%	-9,5%	-9,5%	-7,0%	-6,9%	-10,5%	-8,6%
Switzerland	1674	-6,4%	-14,5%	-10,1%	-16,9%	-12,8%	-7,9%	-8,0%	-13,0%	-7,7%
United Kingdom	4057	-7,7%	-19,0%	-12,2%	-22,5%	-11,7%	-15,2%	-9,6%	-16,5%	-9,6%
United States	5898	-4,7%	-19,4%	-14,2%	-20,9%	-14,5%	-17,6%	-12,9%	-17,6%	-10,1%
**All countries**	**40808**	**-7,4%**	**-15,4%**	**-12,3%**	**-20,2%**	**-12,5%**	**-10,6%**	**-9,2%**	**-14,4%**	**-10,0%**
